# Effects of exogenous silicon on maize seed germination and seedling growth

**DOI:** 10.1038/s41598-020-79723-y

**Published:** 2021-01-13

**Authors:** Yankun Sun, Jiaqi Xu, Xiangyang Miao, Xuesong Lin, Wanzhen Liu, Hongyu Ren

**Affiliations:** College of Resources and Environment, Northeast Agricultural University, Harbin, 150030 China

**Keywords:** Ecology, Physiology, Plant sciences

## Abstract

As the global population continues to increase, global food production needs to double by 2050 to meet the demand. Given the current status of the not expansion of cultivated land area, agronomic seedlings are complete, well-formed and strong, which is the basis of high crop yields. The aim of this experiment was to study the effects of seed germination and seedling growth in response to silicon (from water-soluble Si fertilizer). The effects of Si on the maize germination, seedling growth, chlorophyll contents, osmoprotectant contents, antioxidant enzyme activities, non-enzymatic antioxidant contents and stomatal characteristics were studied by soaking Xianyu 335 in solutions of different concentrations of Si (0, 5, 10, 15, 20, and 25 g·L^−1^). In this study, Si treatments significantly increased the seed germination and per-plant dry weight of seedlings (P < 0.05), and the optimal concentration was 15 g·L^−1^. As a result of the Si treatment of the seeds, the chlorophyll content, osmotic material accumulation and antioxidant defence system activity increased, reducing membrane system damage, reactive oxygen species contents, and stomatal aperture. The results suggested that 15 g·L^−1^ Si significantly stimulated seed germination and promoted the growth of maize seedlings, laying a solid foundation for subsequent maize growth.

## Introduction

Maize, an annual herbaceous plant species, is one of the most important crop species and is a major source of feed and industrial raw materials^1^. Maize plays an important role in ensuring food security and social and economic stability. In recent years, due to the increasing frequency of extreme weather events, unstable yields and low quality of maize caused by seed germination problems in the cold regions of northern China have become an important hidden danger of China's food security^2^. Seeds are the result of survival mechanisms of plants and constitute the material basis for production. The growth status during seed germination directly affects the growth of plants^3^. The rapid germination of maize seeds after sowing leads to early-stage seedlings, homogeneous seedlings and robust seedlings, which constitutes the solid foundation for producing high-quality and high-yield of crops^4^.

As a basic element of animals and humans, Si is a beneficial but unnecessary element of higher plants^4,5^. Although the importance of Si to plants has not been shown, its beneficial role in promoting plant growth has been demonstrated in laboratory and field experiments^6^. Si can enhance seed vigor, increase seedling relative growth rates, increase seedling dry weight, and promote seedling cotyledon growth, laying a foundation for increasing yield and improving quality^7–10^.

Si is listed as the fourth element after nitrogen (N), phosphorus (P) and potassium (K) in the international soil classification system. Although soils are rich in Si, most Si cannot be fully absorbed by plants^11^. At present, there are two main types of Si fertilizer in China: citrate-soluble and water-soluble Si fertilizer. Citrate-soluble Si fertilizer is insoluble in water but soluble in acid; it can be absorbed by crops immediately after application. Citrate-soluble Si typically is a waste product of steel slag during steelmaking, fly ash, or ore through high-temperature calcination process, etc. However, because the raw material is mainly industrial waste and the manufacturing process is relatively simple; many toxic substances from industrial production remain in this type of fertilizer. Water-soluble Si fertilizer is soluble in water and can be directly absorbed and utilized by plants. It is synthesized by high-temperature chemical reactions from a complex production technique that is expensive and produces a relatively small amount^12^.

Si-50G and Si-60G are new water-soluble Si fertilizers synthesized by sol method. The Si-50G and Si-60G have been reported to increase the yield of rice, the state of soil Si and the soil available nutrient content under low temperature stress^13,14^. Although several studies have shown that Si treatment can promote rice growth, its effects on maize seed germination and growth are poorly understood. The objective of this work was to evaluate the effects of different concentrations of Si (from water-soluble Si fertilizer) on seed germination and seedling growth. The reaction was evaluated on the basis of the germination index, growth index, chlorophyll content, peroxidase content, antioxidant enzyme activity, non-enzymatic antioxidant content and stomatal characteristics. With this information, we can better understand the possible mechanisms by which Si promotes maize seed germination and seedling growth to select the best type and concentration, providing a scientific basis for Si application in maize production.

## Results

### Effects of Si on the germination indices

As shown in Table 1, the Si-50G treatments (5 ~ 25 g·L^−1^) and the Si-60G treatments (5 ~ 20 g·L^−1^) could promote the germination of maize seeds, resulting in GR, GP, GI and VI values that were higher than those in the control group. However, Si-60G application decreased the GR, GI and VI in the 25 g·L^−1^ treatments. Compared to those of the control, the GR, GP, GI and VI under a concentration of 15 g·L^−1^ (Si-50G) markedly increased by 19.32%, 38.89%, 53.70% and 129.01%, respectively (P < 0.01). With the increase of concentration, the content of Si first increased but then decreased, and the effects were the most obvious at the concentration of 15 g·L^−1^.

**Table 1:** Effects of the Si (5, 10, 15, 20, and 25 g·L^−1^) on germination indices.

Type	Concentration (g·L^-1^)	GR (%)	GP (%)	GI	VI	Si content (mg·g^−1^)
Control	0	82.00 ± 2.00e	44.00 ± 2.00e	11.78 ± 0.66d	9.36 ± 0.77g	0 ± 0c
Si-50G	5	93.63 ± 0.61b	50.00 ± 3.33cd	16.75 ± 0.36b	16.97 ± 0.46c	6.03 ± 0.7ab
	10	96.01 ± 0.57a	53.73 ± 0.53bc	16.99 ± 0.82b	17.85 ± 1.24c	6.06 ± 0.57ab
	15	97.84 ± 1.87a	61.11 ± 3.85a	18.11 ± 0.86a	21.43 ± 1.18a	6.67 ± 0.84a
	20	97.06 ± 0.67a	57.78 ± 1.92ab	17.01 ± 0.71b	19.26 ± 0.52b	6.56 ± 0.5a
	25	88.89 ± 2.22c	44.00 ± 2.65e	13.07 ± 0.49c	12.93 ± 0.51e	5.76 ± 0.52ab
Si-60G	5	84.00 ± 1.00de	55.33 ± 2.31b	13.62 ± 0.78c	11.37 ± 0.68f	5.4 ± 0.31bc
	10	88.00 ± 2.00c	60.00 ± 2.00a	13.63 ± 0.19c	13.36 ± 0.02de	5.75 ± 0.78ab
	15	90.33 ± 0.58c	60.67 ± 1.53a	13.85 ± 0.16c	14.33 ± 0.25d	5.92 ± 0.62ab
	20	85.33 ± 0.58d	49.33 ± 2.08d	13.46 ± 0.22c	12.17 ± 0.34ef	5.87 ± 1.02ab
	25	76.67 ± 1.53f	42.22 ± 1.92e	11.62 ± 0.39d	8.85 ± 0.54g	4.74 ± 0.52c

The values represent the mean ± SE (n = 5). Values with the same letters in the columns are not significantly different at P < 0.05.

### Effects of Si on seedling growth

With the increase of concentration, the growth indices of the seedlings first increased but then decreased, and the effects were the most obvious at the concentration of 15 g·L^−1^ (Table 2). Compared with those of the control, the shoot height, root height, shoot fresh weight, root fresh weight, shoot dry weight, root dry weight under Si-50G 15 g·L^−1^ increased by 22.66%, 22.90%, 51.93%, 62.40%, 45.83%, and 50.00%, respectively; furthermore, compared with those of the control, increased by 9.10%, 13.24%, 45.01%, 51.55%, 18.75% and 46.88% under the Si-60G 15 g·L^−1^, respectively. Moreover, compared with those of the control, the shoot height, root height, shoot fresh weight, root fresh weight, shoot dry weight under the Si-60G 25 g·L^−1^ decreased by 9.04%, 2.46%, 2.24%, 3.10% and 6.25%, respectively. Compared with that of the control, the Si content under 15 g·L^−1^ increased by 4.69% and 4.38% (P < 0.01).

**Table 2:** Effects of Si (5, 10, 15, 20, and 25 g·L^−1^) on seedling growth.

Type	Concentration (g·L^-1^)	Shoot height/(cm)	Root length (cm)	Shoot fresh mass (g·plant^−1^)	Root fresh mass (g·plant^−1^)	Shoot dry weight (g·plant^−1^)	Root dry weight of (g·plant^−1^)	Si content (mg·g^−1^)
Control	0	57.50 ± 0.36i	27.20 ± 0.40f	4.91 ± 0.01h	2.58 ± 0.01f	0.48 ± 0.01f	0.32 ± 0.01g	4.23 ± 0.09bc
Si-50G	5	65.47 ± 0.31d	30.83 ± 0.42c	6.21 ± 0.07e	3.48 ± 0.26d	0.60 ± 0.02b	0.42 ± 0.01e	4.35 ± 0.05ab
	10	67.50 ± 0.30c	31.83 ± 0.06b	6.72 ± 0.09cd	3.66 ± 0.08c	0.62 ± 0.01b	0.43 ± 0.01d	4.36 ± 0.06a
	15	70.53 ± 0.31a	33.43 ± 0.32a	7.46 ± 0.15a	4.19 ± 0.01a	0.70 ± 0.02a	0.48 ± 0.01a	4.43 ± 0.06a
	20	68.33 ± 0.25b	31.80 ± 0.70b	6.84 ± 0.17bc	3.68 ± 0.10c	0.68 ± 0.02a	0.45 ± 0.01bc	4.40 ± 0.06a
	25	63.63 ± 0.25e	30.80 ± 0.35c	5.89 ± 0.23f	3.34 ± 0.05d	0.59 ± 0.01b	0.40 ± 0.01f	4.34 ± 0.06ab
Si-60G	5	59.23 ± 0.35h	29.47 ± 0.31d	6.30 ± 0.07e	3.30 ± 0.07d	0.50 ± 0.02e	0.33 ± 0.01g	4.35 ± 0.06ab
	10	60.50 ± 0.30g	29.83 ± 0.35d	6.47 ± 0.36de	3.42 ± 0.03d	0.54 ± 0.01d	0.44 ± 0.01cd	4.35 ± 0.06ab
	15	62.73 ± 0.61f	30.80 ± 0.40c	7.12 ± 0.27b	3.91 ± 0.16b	0.57 ± 0.01c	0.47 ± 0.01ab	4.42 ± 0.05a
	20	59.40 ± 0.35h	28.40 ± 0.46e	5.55 ± 0.12g	2.78 ± 0.06e	0.50 ± 0.01ef	0.41 ± 0.01ef	4.37 ± 0.07a
	25	52.30 ± 0.46j	26.53 ± 0.31f	4.80 ± 0.14h	2.50 ± 0.06f	0.45 ± 0.01g	0.32 ± 0.01g	4.21 ± 0.07c

The values represent the mean ± SE (n = 4). Values with the same letters in the columns are not significantly different at P < 0.05.

### Effects of Si on the photosynthetic pigment contents

The Si-50G treatments (5 ~ 25 g·L^−1^) markedly increased the *Chl a* + *b* content, the *Chl a* content, the *Chl b* content, and the *Car* content by 0.92% ~ 6.81%, 1.49% ~ 5.58%, 0.29% ~ 8.27%, and 3.09% ~ 6.99%, respectively (Table 3). The Si-60G treatments (5 ~ 20 g·L^−1^) markedly increased the *Chl a* + *b* content, the *Chl a* content, the *Chl b* content, and the *Car* content by 0.27% ~ 3.30%, 0.60% ~ 4.48%, 0.29% ~ 1.98%, and 2.87% ~ 5.88%, respectively. Compared with those of control, *Chl a* + *b*, *Chl a* , *Chl b* and *Car* contents under the Si-60G 25 g·L^−1^ decreased by 1.91%, 1.42%, 2.88% and 2.21%, respectively.

**Table 3:** Effects of Si (5, 10, 15, 20, and 25 g·L^−1^) on the photosynthetic pigment contents.

Type	Concentration (g·L^-1^)	*Chl* a + b (mg·g^-1^FW)	*Chl* a (mg· g^-1^FW)	*Chl* b (mg·g^-1^FW)	*Car *(mg·g^-1^FW)
Control	0	5.24 ± 0.06cd	2.81 ± 0.05cd	2.43 ± 0.11bc	1.36 ± 0.06de
Si-50G	5	5.33 ± 0.18bc	2.87 ± 0.07abcd	2.46 ± 0.13bc	1.40 ± 0.02cd
	10	5.37 ± 0.11bc	2.91 ± 0.08abc	2.46 ± 0.08bc	1.44 ± 0.01abc
	15	5.60 ± 0.03a	2.97 ± 0.07a	2.63 ± 0.07a	1.46 ± 0.03a
	20	5.49 ± 0.10ab	2.95 ± 0.04a	2.53 ± 0.11ab	1.45 ± 0.01ab
	25	5.29 ± 0.08cd	2.86 ± 0.06abcd	2.44 ± 0.09bc	1.40 ± 0.01bcd
Si-60G	5	5.26 ± 0.03cd	2.81 ± 0.05bcd	2.43 ± 0.06bc	1.40 ± 0.01cd
	10	5.37 ± 0.12bc	2.91 ± 0.06abc	2.46 ± 0.05bc	1.42 ± 0.02abc
	15	5.42 ± 0.06bc	2.94 ± 0.07ab	2.48 ± 0.02bc	1.44 ± 0.01 abc
	20	5.30 ± 0.06cd	2.86 ± 0.07abcd	2.44 ± 0.03bc	1.43 ± 0.01abc
	25	5.14 ± 0.08d	2.78 ± 0.04d	2.36 ± 0.04c	1.33 ± 0.04e

The values represent the mean ± SE (n = 4). Values with the same letters in the columns are not significantly different at P < 0.05.

### Effects of Si on osmolyte contents

As shown in Table 4, except for the sugar and proline contents under the Si-60G 25 g·L^−1^, the soluble sugar, soluble protein and proline contents in maize leaves significantly increased with Si application compared with the control (P < 0.05). Compared with those of the control, the soluble sugar, soluble protein and proline contents under the Si-50G at 15 g·L^−1^ significantly increased by 23.89%, 34.07% and 27.27%, respectively (P < 0.01); furthermore, compared with those of the control, the same parameters under the Si-60G at 15 g·L^−1^ increased by 20.69%, 31.42% and 20.13%, respectively (P < 0.01). However, the soluble sugar and proline contents decreased by 2.46% and 1.32%, respectively, and the soluble protein contents significantly decreased by 5.75% compared with the control values.

**Table 4:** Effects of Si (5, 10, 15, 20, and 25 g·L^−1^) on on the contents of osmolytes (sugar, protein and proline).

Type	Concentration (g·L^−1^)	Sugar content (mg·g^−1^ FW)	Protein content (mg·g^−1^ FW)	Proline content (μg·g^−1^ FW)
CK	0	4.06 ± 0.09f	2.26 ± 0.04h	3.08 ± 0.13e
Si-50G	5	4.27 ± 0.04e	2.53 ± 0.08f	3.34 ± 0.03d
	10	4.33 ± 0.03de	2.63 ± 0.08de	3.70 ± 0.06d
	15	5.03 ± 0.03a	3.03 ± 0.04a	3.92 ± 0.03a
	20	4.44 ± 0.07d	2.88 ± 0.06b	3.33 ± 0.09d
	25	4.26 ± 0.04e	2.40 ± 0.08g	3.24 ± 0.03d
Si-60G	5	4.26 ± 0.03e	2.55 ± 0.04ef	3.31 ± 0.08d
	10	4.85 ± 0.04bc	2.77 ± 0.02c	3.62 ± 0.01b
	15	4.90 ± 0.07ab	2.97 ± 0.06ab	3.70 ± 0.03b
	20	4.73 ± 0.24c	2.67 ± 0.06*d	3.49 ± 0.01c
	25	3.96 ± 0.02f	2.13 ± 0.04i	3.04 ± 0.07e

The values represent the mean ± SE (n = 4). Values with the same letters in the columns are not significantly different at P < 0.05.

### Effects of Si on reactive oxygen species

**Figure 1:**
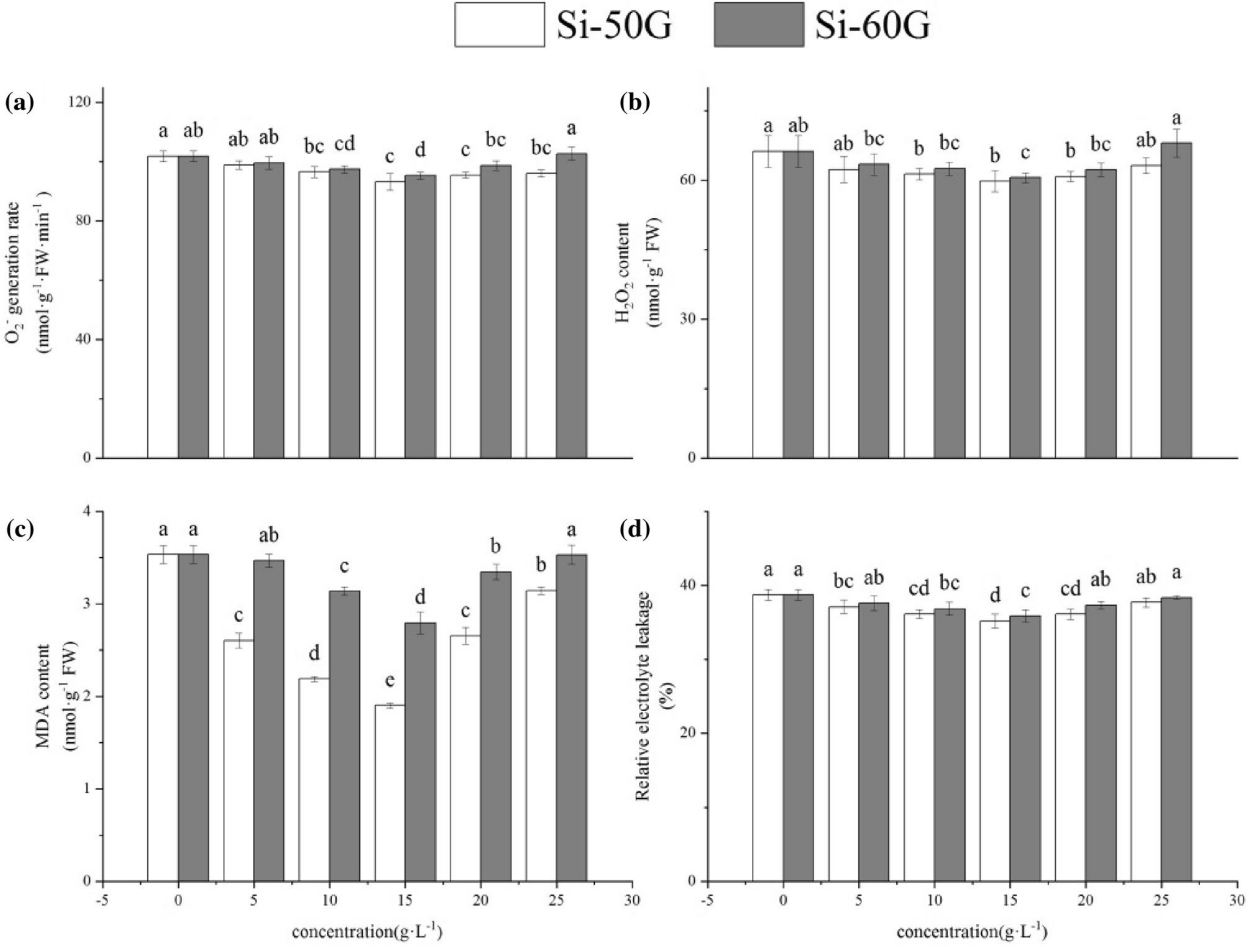
Effects of Si (5, 10, 15, 20, and 25 g·L^−1^) on ROS and lipid peroxidation. The values represent the mean ± SE (n = 4). Mean values provided with error bars representing the standard error. Different letters denote statistical differences at P < 0.05.

Si reduced the generation rate of O_2_^·−^ and H_2_O_2_ content in seedlings, and the effects were more pronounced under 15 g·L^−1^ (Fig. 1a,b). Compared with the control values, the Si-50G treatments decreased the generation rate of O_2_^·−^ by 2.90%, 5.20%, 8.50%, 6.28% and 5.67% and decreased the H_2_O_2_ content by 5.90%, 7.41%, 9.70%, 8.20% and 4.63%, respectively. Moreover, the Si-60G at 5 ~ 20 g·L^−1^ treatments decreased the generation rate of O_2_^·−^ by 2.19%, 4.29%, 6.38% and 3.04%, respectively, and decreased the H_2_O_2_ content by 4.23%, 5.61%, 8.58% and 5.98%, respectively. However, the Si-60G at 25 g·L^−1^ treatment increased the generation rate of O_2_^·−^ and the H_2_O_2_ content by 0.94% and 2.70%, respectively.

### Effects of Si on lipid peroxidation

The non-Si-treated maize seedlings had a higher membrane permeability and MDA content. However, these parameters nearly significantly decreased compared with those of the control when Si was present. Compared with those of the control, the MDA content and EL under the Si-50G 15 g·L^−1^ significantly decreased by 46.17% and 20.93% (Fig. 1c) and by 9.17% and 7.36% under the Si-60G 15 g·L^−1^ (Fig. 1d), respectively (P < 0.01).

### Effects of Si on antioxidant enzyme activities

**Figure 2:**
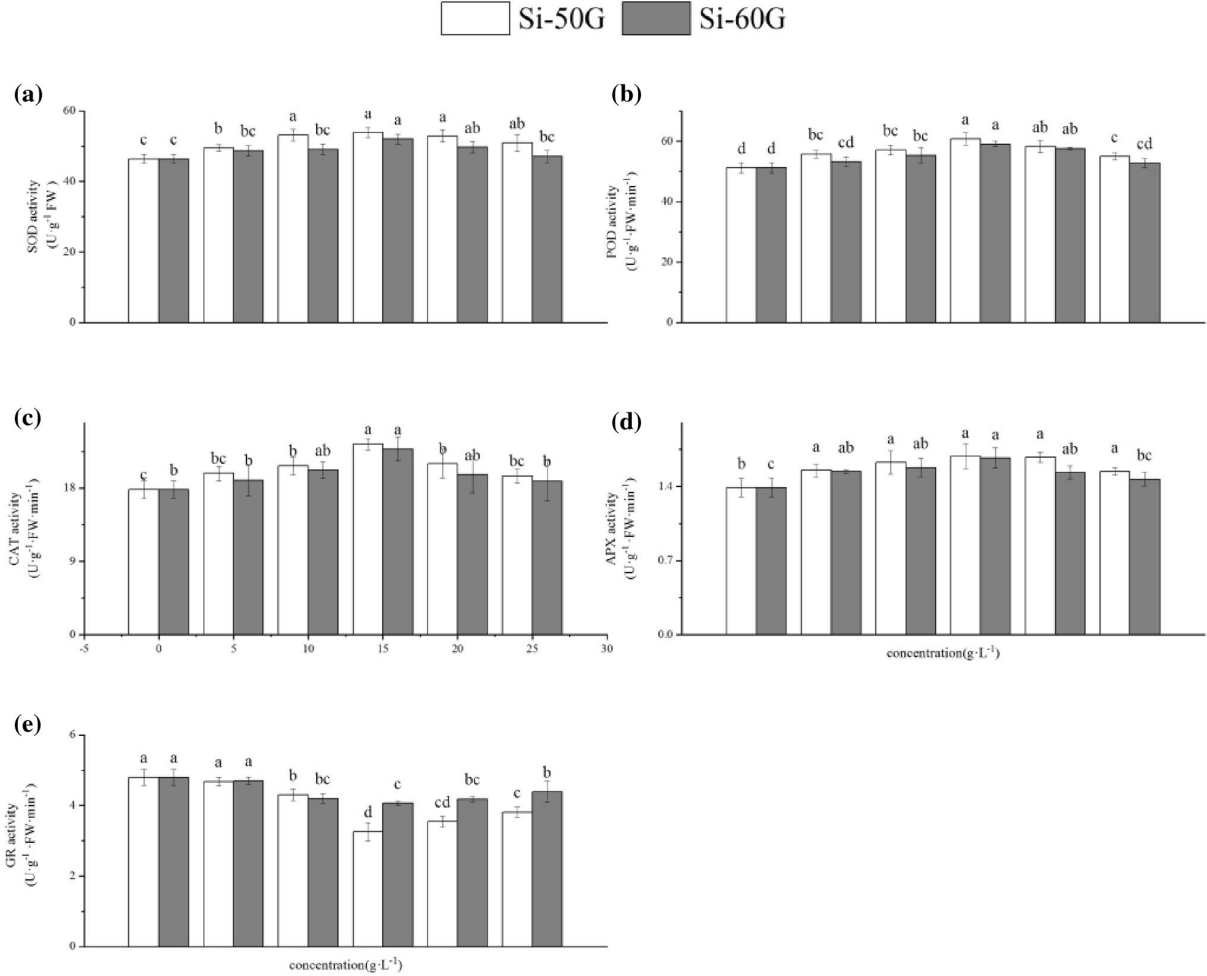
Effects of Si (5, 10, 15, 20, and 25 g·L^−1^) on antioxidant enzyme activities. The values represent the mean ± SE (n = 4). Mean values provided with error bars representing the standard error. Different letters denote statistical differences at P < 0.05.

Compared with those of the control, the activities of SOD, POD, CAT and APX in the leaves in response to Si application increased, and the GR activity decreased (Fig. 2). Compared with that of the controls, the SOD, POD, CAT and APX increased by 16.16%, 12.08%, 18.73% and 15.38%, respectively, and by 31.31% and 27.89%, 21.31% and 20.06%, respectively at the 15 g·L^−1^(P < 0.01). However, compared with that of the control, the GR activity was decreased by 32.21% and 18.12%, respectively at the 15 g·L^−1^ (P < 0.01) (Fig. 2e).

### Effects of Si on non-enzymatic antioxidant contents

**Figure 3:**
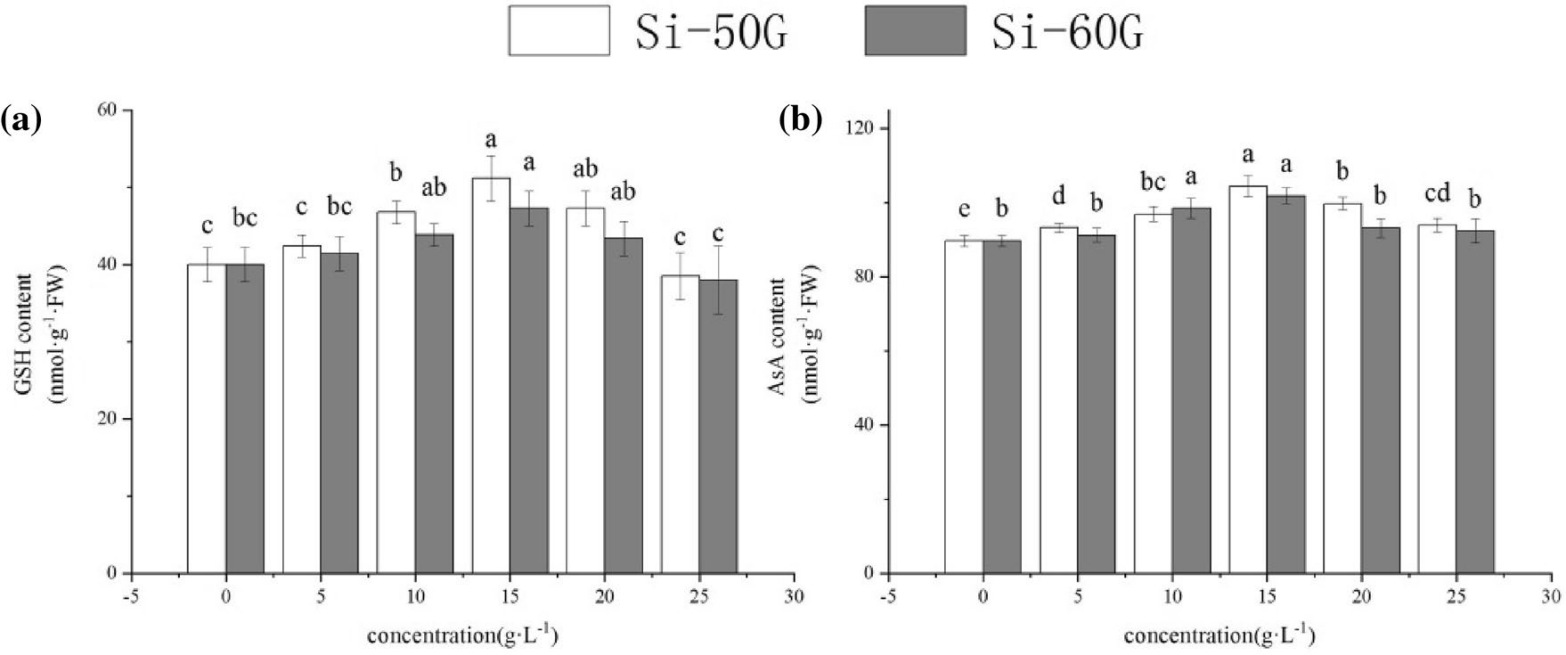
Effects of Si (5, 10, 15, 20, and 25 g·L^−1^) on on non-enzymatic antioxidant contents. The values represent the mean ± SE (n = 4). Mean values provided with error bars representing the standard error. Different letters denote statistical differences at P < 0.05.

Compared with those of the control, the GSH and AsA contents under Si-50G at 15 g·L^−1^ clearly increased by 28.05% and 18.29%, respectively (Fig. 3a); furthermore, compared with those of the control, the same parameters in the Si-60G at 15 g·L^−1^ treatment group increased by 16.46% and 13.50%, respectively (P < 0.01) (Fig. 3b).

### Effects of Si on stomatal size and aperture

The size of stomatal notably changed. With the increasing Si concentration, the length and width of the stomata and the stomatal aperture first increased but then decreased. However, with the increasing concentration, the width of stomatal aperture first decreased but then increased. In addition, under the 15 g**·**L^−1^, the width of stomatal aperture significantly decreased by 24.34% and 17.11% (P < 0.01), respectively, compared with those of the control (Table 5).

**Table 5:** Effects of Si (5, 10, 15, 20 or 25 g·L^−1^) on stomatal size and aperture.

Type	Concentration (g·L^-1^)	Stomatal size(μm)		Stomatal aperture(μm)		
		Length	Width	Length		Width
Control	0	30.73 ± 0.25f	22.90 ± 0.70ab	17.50 ± 0.10g		1.52 ± 0.02a
Si-50G	5	28.33 ± 0.21g	16.00 ± 0.20f	17.97 ± 0.29g		1.32 ± 0.03bc
	10	28.97 ± 0.45g	19.07 ± 0.51e	19.30 ± 0.10f		1.26 ± 0.06c
	15	36.30 ± 0.70c	23.33 ± 0.65a	27.43 ± 0.12a		1.15 ± 0.05d
	20	34.77 ± 0.31d	21.67 ± 1.10bc	22.60 ± 0.26c		1.30 ± 0.03bc
	25	30.97 ± 0.40f	21.47 ± 0.91cd	20.23 ± 0.47e		0.79 ± 0.03e
Si-60G	5	34.23 ± 0.50d	20.03 ± 0.40e	19.90 ± 0.01ef		1.38 ± 0.05b
	10	38.30 ± 0.17b	20.30 ± 0.86de	21.80 ± 0.89d		1.35 ± 0.10bc
	15	40.50 ± 0.30a	23.70 ± 0.90a	23.70 ± 0.35b		1.26 ± 0.04c
	20	34.40 ± 0.26d	19.57 ± 0.81e	22.73 ± 0.68c		1.32 ± 0.08bc
	25	33.43 ± 0.25e	13.77 ± 0.49g	19.70 ± 0.66ef		0.70 ± 0.01f

The values represent the mean ± SE (n = 4). Values with the same letters in the columns are not significantly different at P < 0.05.

## Discussion

The GR, GP, GI and VI of seeds are important indicators to measure the germination performance of plant seeds^15^. Seed germination and seedling emergence are the most critical and sensitive stages in plant life cycle. The results of this study show that the Si-50G treatments (5 ~ 25 g·L^−1^) and the Si-60G treatments (5 ~ 20 g·L^−1^) could significantly improve the GR, GP, GI and VI of maize seeds (Table 1). This indicates that the appropriate concentration of Si can help maize seeds break dormancy, accelerate the restoration of seed vigor and recovery from damage when drying seeds, complete the germination and emergence process, and provide better physiological conditions for subsequently seedling growth^2^.

Exogenous Si can promote the growth of soybean^16^, barley^17^ and seedlings of other species. Moreover, exogenous Si can increase the root length and seedling height and can increase the fresh and dry weight of seedlings. In this study, most of the Si treatments enhanced the height, length and dry weight of maize seedlings (Table 2). The highest germination percentage was obtained under the Si-50G at 15 g·L^−1^, and the total dry and fresh weight of seedlings significantly increased. The effects of 15 g·L^−1^ Si on the maize root system was better than that on the whole plants. The establishment of seedlings after seed germination is the period that is most sensitive to the external environment. A healthy and intact root system can ensure that plants can absorb sufficient amounts of water, nutrients and mineral ions from the soil and promote photosynthesis of plant leaves to complete self-growth^2^.

*Chl a* and *Chl b* are the main pigments that absorb and transmit light energy, which can improve the efficiency of light capture^18^. *Car* is photosynthetic pigment that absorbs light energy and transmits it to the reaction centers. Our results suggested that by 15 g·L^−1^ Si could increase the contents of *Chl a* + *b*, *Chl a*, *Chl b* and *Car* (Table 3). The application of Si may be associated with an increase in chlorophyll content, which may be because Si can eventually enter into the chloroplast through plant absorption and transport, increasing the chlorophyll content of the plant and thus increasing photosynthesis^19,20^. However, the Si-60G at 25 g·L^−1^ reduced the contents of *Chl a* + *b, Chl a, Chl b* and *Car* in maize seedlings. This may be due to the excessive concentration of Si, which reduces chlorophyll synthesis, while a decrease of soluble sugar content both inhibits stomatal opening and normal photosynthesis^21^.

As osmotic regulators, soluble sugar and proline can effectively reduce the osmotic potential of plant cells by increasing its content^22,23^. The osmotic function of soluble protein is well known^24^. Si increased the content of soluble sugar, soluble protein and proline in maize seedlings (Table 4) and decreased the cell fluid osmotic potential (Fig. 2d), indicating that Si could play an important regulatory role in maintaining normal cell metabolism. In addition, according to the above experiments, Si can promote the synthesis of soluble sugar, soluble protein and proline, maintain its contents at a high level, and increase the ability of osmotic regulatory ability of seedlings. With applications of Si, the increase in these permeable substances may be an effective mechanism to improve the ability of plants to resist oxidative stress^25^.

Under adverse conditions, plants produce an excess amount of reactive oxygen species (ROS)^26^, which can cause membrane peroxidation and electrolyte leakage in cells, eventually destroying the structure and function of the cell membrane^27–29^. However, the protective system of the organisms can remove these ROS and reduce harm. SOD, POD, CAT, APX and GR are the main enzymes involved in the protection system, and these enzymes play a key role in enhancing plant stress resistance and maintaining normal plant growth, metabolism and development^30^. Changes in the activities of these enzymes reflect the protective response of plants. SOD is a purifier of O_2_^·−^ and protects cells by removing O_2_^·−^ and reducing the concentration of other reactive oxygen species produced by O_2_^·−^. POD is a protective enzyme with high activity that is widely present in plants; it plays an important role in cell metabolism. The increase in POD activity is beneficial to plant growth and development^31^. CAT can remove H_2_O_2_ produced by stress in plants and decompose the accumulated H_2_O_2_ into H_2_O and O_2_, thus reducing its ability to damage plant tissues oxidatively^32^. The enzymatic antioxidants APX and GR, as well as non-enzymatic antioxidants AsA and GSH, are important components to eliminate ROS and reduce oxidative damage under abiotic stress^33^. These antioxidants have their own functions and maintain the redox state of cells^34,35^. In this study, the appropriate concentration of Si could enhance the activity of SOD, POD, CAT and APX and the content of AsA and GSH; reduce the content of MDA; effectively enhance the ROS-scavenging ability of plants to maintain the balance of ROS metabolism; reduce oxidative stress reactions; and reduce the degree of cell EL, thus promoting the growth of maize^10,36^. However, the Si-60G at 25 g·L^−1^ treatment resulted in an increase in the content of MDA and EL, which may be related to the accumulation of O_2_^·−^ and H_2_O_2_, leading to damage to the biofilm system, and thus inhibiting the growth of seedlings (Table 2)^29^. Si can affect the accumulation of ROS in plants, help maintain the balance of reactive oxygen metabolism, reduce membrane peroxidation, maintain the integrity of the membrane system, and enhance the activity of enzymes involved in the antioxidant system, thus promoting plant growth.

**Figure 4:**
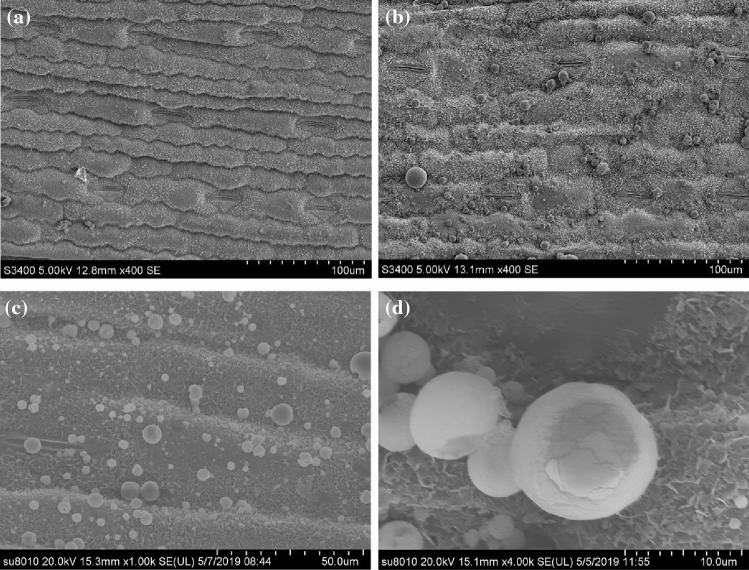
Stomata on a maize leaf. (**a**) shows stomata on the abaxial side; (**b**) shows stomata on the adaxial side; (**c**) magnified 1000 times of small spheres around the stomata of the adaxial leaf side; (**d**) magnified 1000 times of small spheres around the stomata of the adaxial leaf side.

**Figure 5:**
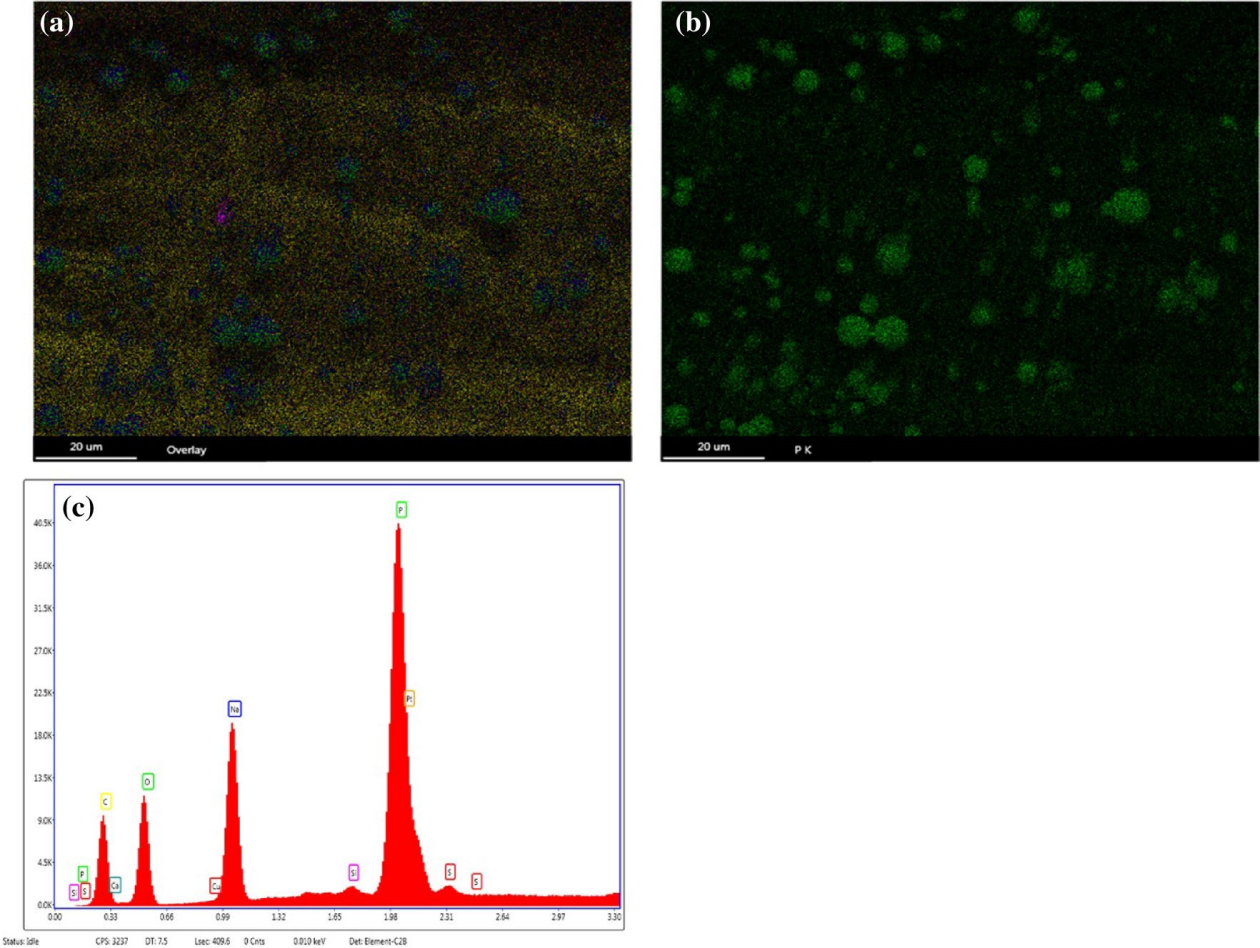
(**a**) shows the distribution of the main elements on the surface of the sphere; (**b**) shows the distribution of P; (**c**) shows the elements detected on the surface of the spheres by EDS.

Stomatal size affects plant water-use efficiency, photosynthetic rate and yield^37^. The stomata control the exchange of water vapor and carbon dioxide between the blade's interior and the atmosphere. The adjustment of the stomatal aperture can affect the progress of photosynthesis in plants^38^. In this study, Si reduced the stomatal width and facilitates stomatal opening and closing (Table 5). These findings are consistent with those from Laza's research on rice^39^. Many small spheres with a metallic luster were found around the stomata on the adaxial side of leaves under the Si-60G treatment at a concentration of 15 g·L^−1^ (Fig. 4b–d), compared with the stomata on the abaxial side (Fig. 4a). The element distribution on the surface of the sphere was subsequently analysed by energy dispersive spectrometry (EDS) (Fig. 5c). The distribution of the main elements on the surface of the spheres was shown (Fig. 5a), and many P elements were found on the surface of the spheres (Fig. 5b), which corroborates the findings of other researchers^40,41^. The P uptake of maize in response to Si fertilizer significantly increased, and there was a significant positive correlation between P uptake and Si uptake of maize. In the future, we need to further determine the route of P transport in maize and study the mechanism by which Si alleviates drought stress.

## Conclusion

The present results showed that exogenous Si can improve the seed germination rate, germination potential, germination index and vigor index; promote seedling growth; and increased chlorophyll contents. In addition, Si could improve the antioxidant defence ability of maize plants and increase the contents of osmotic substances, thereby increasing the ability to remove ROS and maintain the integrity of the membrane system. Among the Si treatments, Si exerted the best positive effects at 15 g·L^−1^, while negative effects occurred in response to 25 g·L^−1^ (Si-60G) these findings indicate that the appropriate concentration of exogenous Si can promote maize seed germination and seedling growth laying a good foundation for subsequent growth.

## Materials and methods

### Plant material and treatments

**Figure 6:**
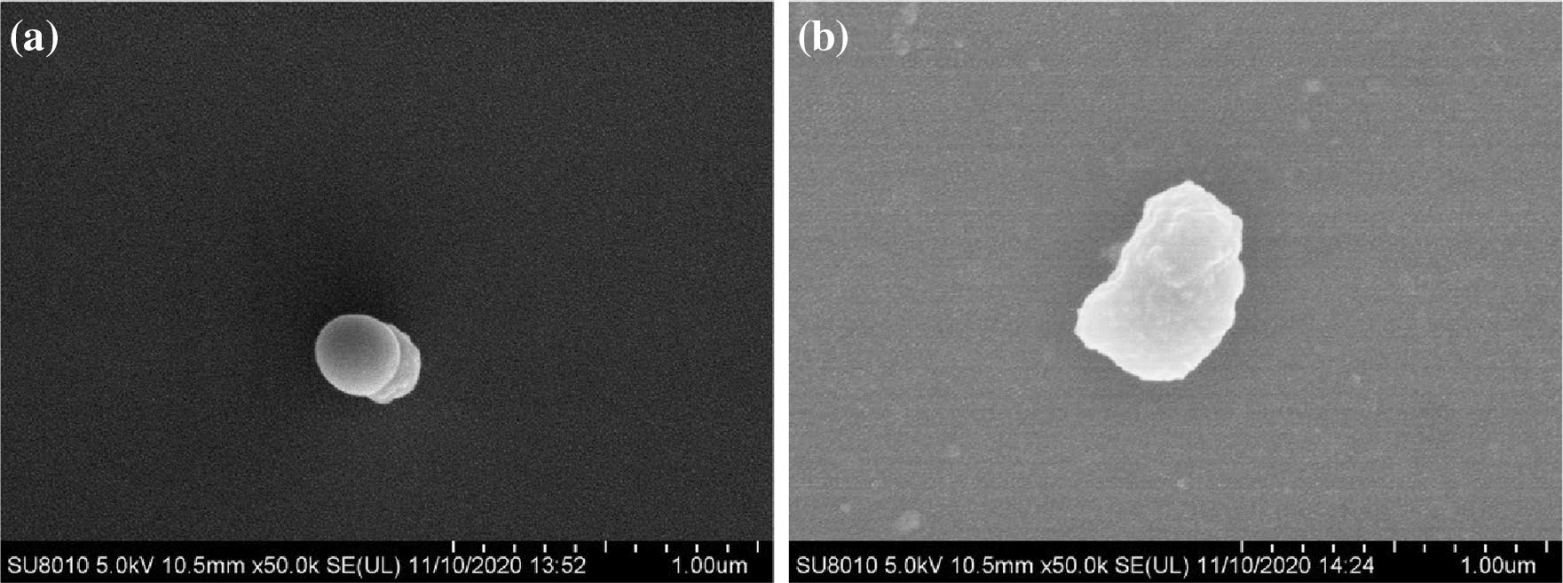
Scanning electron microscope images of Si preparation. (**a**) shows Si-50G. (**b**) shows Si-60G.

This experiment was conducted in a controlled growth chamber at the Northeast Agricultural University of Harbin, Heilongjiang Province, China (45° 448′ 52″ N, 126° 43′ 9″ E). Exogenous Si compounds were provided by the school of Chemistry and Chemical Engineering, Harbin Institute of Technology. The characteristic features of Si preparation given in Table 6 and the particle size of Si preparation is shown in Fig. 6. The tested maize cultivar was Xianyu 335. Healthy, plump and pest-free maize seeds were sterilized for 20 min with 30% (w/v) hydrogen peroxide (H_2_O_2_), and then repeatedly rinsed and soaked abundantly with distilled water. Afterward, we wiped the water off the maize seeds surface with a filter and set the seeds aside. Five replicates (thirty seeds each) were placed on two layers of filter paper in 9 cm diameter petri dishes containing 15 mL of distilled water (control), and different concentration of exogenous Si (5, 10, 15, 20, and 25 g·L^−1^). The seeds were then allowed to germinate under the following environmental conditions: average day/night temperature of 25/18 °C, relative humidity of 65 ± 5%, light intensity of 350 μmol·m^−2^·s^−1^ and photoperiod of 16 h. To ensure the relative stability of each treatment concentration, the solution was supplemented once every 24 h and the number of maize seeds that germinated was recorded once every 24 h.

**Table 6:** Physico-chemical properties of Si preparation.

Properties	Types	
	Si-50G	Si-60G
Raw material	3-aminopropyl triethoxy silane	3-glyci doxypropyl trimethoxy silane
Preparation method	Sol—gel process	Sol–gel process
pH	5.95	9.42
Morphology	Spherical	Irregular
Average particle size	323 nm

After the germination (for 7 days), relatively uniform seedlings were selected and transferred to opaque plastic pots (inner diameter, 15.5 cm; external diameter, 18 cm; and height, 11 cm) filled with 1.25 kg of soil per pot under the same environmental conditions as those reported above. The soil used in the experiment was black soil. The physical and chemical properties of the soil were as follows: pH, 8.06; organic matter, 46.52 g·kg^−1^; total nitrogen, 3.58 g·kg^−1^; total phosphorus, 1.21 g·kg^−1^; total potassium, 182.7 mg·kg^−1^; available phosphorus, 51.39 mg·kg^−1^; available nitrogen, 144.90 mg·kg^−1^; and Si, 78.8 mg·kg^−1^. Maize seedlings at the three-leaf stage were used for the treatments with concentrations (5, 10, 15, 20 and 25 g·L^−1^) of Si and distilled water (control). The seedlings were sprayed once every 3 days, for a total of 2 times. Five millilitres was sprayed onto both sides of each leaf. Each treatment was repeated 4 times for a total of 44 pots.

Whole plants were sampled from each treatment on the 7th day for measurements of the growth parameters. The second fully developed leaves of the seedlings were removed on the 7th day for measurements of the growth parameters. The second fully developed leaves of the seedlings were removed on the 7th day for scanning electron microscope observations and measuring the electrolyte leakage (EL). The remaining leaves were immediately frozen in liquid nitrogen and stored at − 80 °C for subsequent determination of the other indicators^42^.

### Measurements of germination

Seeds were considered to have germinated when the radicle extended for at least 2 mm. After seven days of germination, the germination rate (GR), germination potential (GP), germination index (GI) and vigour index (VI) were measured according to following Eqs.^10^:

1 $$ {\text{GR}} = {\text{n}}/{\text{N}} \times {1}00\% $$

where, n is the number of germinated seeds and N represents the total number of seeds tested;

2 $$ {\text{GP}} = {\text{a}}/{\text{N}} \times {1}00\% $$

where, a is the number of germinated seeds after three days;

3 $$ {\text{GI}} = \sum {\text{Gt}}/{\text{Dt}} $$

where, Gt is the number of seeds germinated on t days and Dt represents the corresponding day of germination; and

4 $$ {\text{VI}} = {\text{ GI}} \times {\text{mean of the dry weight of seedlings}} $$

### Measurements of plant growth

The maize seedlings with their roots were collected and rinsed with tap water and distilled water. The maize seedlings were then separated into roots and shoots, which were weighted separately to determine the fresh weight (FW) of each part. The height of the shoots and the length of roots of each plant were simultaneously measured by ruler. Dry weight (DW) was measured after the samples had dried in an oven dried at 105 °C for 30 min and had maintained constant weight at 80 °C for 24 h.

### Measurements of pigment contents

Total chlorophyll, chlorophyll a (*Chl a*), chlorophyll b (*Chl b*) and carotenoid (*Car*) compounds were extracted, after which their contents were measured and quantified according to modified method of Arnon^43^. After the extraction and analysis, the relative contents of *Chl a*, *Chl b*, *Car* and the total content of chlorophyll were calculated according the following formulae:

5 $$ Chl \, a\left( {{\text{mg}}\cdot{\text{g}}^{{ - {1}}} } \right) \, = \, \left[ {\left( {{12}.{\text{71A}}_{{{663}}} - {2}.{\text{59A}}_{{{645}}} } \right){\text{V}}} \right]/{\text{W}} $$

6 $$ Chl \, b\left( {{\text{mg}}\cdot{\text{g}}^{{ - {1}}} } \right) \, = \, \left[ {\left( {{22}.{\text{88A}}_{{{645}}} - {4}.{\text{67A}}_{{{663}}} } \right){\text{V}}} \right]/{\text{W}} $$

7 $$ Car\left( {{\text{mg}}\cdot{\text{g}}^{{ - {1}}} } \right) \, = \, \left( {{1}000{\text{A}}470 - {3}.{27}Chl \, a - {1}0{4}Chl \, b} \right){\text{ V}}/{229}*{\text{W}} $$

8 $$ {\text{Total chlorophyll }}\left( {{\text{mg}}\cdot{\text{g}}^{{ - {1}}} } \right) \, = \, \left[ {\left( {{2}0.{\text{29A}}_{{{645}}} + {8}.0{\text{4A}}_{{{663}}} } \right){\text{V}}} \right]/{\text{W}} $$

where, V represents final volume of chlorophyll extract in 80% acetone and W is 0.1 g.

### Measurement of osmoprotectants

The soluble sugar was quantified by the anthrone sulfuric acid method using 1% anthrone in concentrated H_2_SO_4_ as reagent^44^. Its absorbance was measured at 630 nm, and a standard curve was plotted with 0 ~ 100 mg of glucose.

The soluble protein was measured according to the coomassie brilliant blue G-250 method described by Bradford^45^. Coomassie brilliant blue G-250 (100 mg) was dissolved in 50 mL 95% ethanol. Afterward, 100 mL 85% (w/v) phosphoric acid was added to the solution. The resulting solution was diluted to a final volume of 1 L. The reagent ultimately consisted of 0.01% (w/v) coomassie brilliant blue G-250, 4.7% (w/v) ethanol, and 8.5% (w/v) phosphoric acid, and its absorbance was measured colorimetrically at 595 nm.

Briefly, 100 mg of tissue powder was homogenized in 10 mL of 3% aqueous sulfosalicylic acid for 10 min followed by filtration. Two milliliters of the filtrate, 2 mL of glacial acetic acid and 2 mL of acid ninhydrin were mixed together for 1 h at 90 °C. The sample was extracted in 4 mL toluene and measured colourimetrically at 520 nm against toluene, and a standard curve with proline was used for the final calculations^46^.

### Measurements of reactive oxygen species

First, 0.5 mL of the reaction mixture, 0.5 mL sulfanilic acid solution and 0.5 mL α-naphthylamine solution were mixed together and shaken evenly. After incubating at room temperature for 20 min, the optical density of the mixture was determined at 530 nm via a spectrophotometer^47^.

H_2_O_2_ was extracted by homogenizing 0.3 g leaf tissue with 3 mL of 50 mM phosphate buffer (pH 6.5) together. The homogenate was centrifuged at 6000 rpm for 25 min. The supernatant was brought up to 3 mL with the phosphate buffer. To determine H_2_O_2_ content, 3 mL of extracted solution was mixed with 1 mL of 0.1% titanium sulphate in 20% H_2_SO_4_ (v/v) were mixed together, after which the mixture was then centrifuged at 6,000 rpm for 15 min. The intensity of the yellow color of the supernatant was measured at 410 nm. The H_2_O_2_ content was calculated from a standard curve prepared via hydrogen peroxide of known strength^48^.

### Measurements of lipid peroxidation

Plant samples (0.4 g) were homogenized with 2.4 mL 0.1% trichloroacetic acid (TCA) and the homogenates were centrifuged at 12,000 rpm for 15 min. The supernatant (0.5 mL) and 20% TCA 1:1(v/v) were containing 0.5% (w/v) thiobarbituric acid (TBA) were mixed together (1:1), after which the solution was heated at 100 °C for 20 min. The absorbance was subsequently determined at 450, 532, and 600 nm^25,49^.

EL was used to evaluate plasma membrane permeability and measured using an electrical conductivity meter (DDS-307A, Inasa Analytical Instrument Co., Ltd., Shanghai, P. R. China). Each 0.5 g of fresh leaf material of maize seedling was placed in individual stoppered triangular flasks containing 10 mL of deionized water. The samples were incubated at 25 °C for 24 h. The electrical conductivity of the solution (S_1_) was measured after incubation. The samples were then placed in a boiling water bath for 10 min and the second measurement (S_2_) was determined after the solutions cooled to room temperature. EL = S_1_/S_2_ × 100%^50^.

### Measurements of antioxidants

Crude enzyme extracts were prepared according to the method described by Pan et al*.*^51^. A total of 0.5 g of frozen leaf samples from maize seedlings were ground to a fine powder with liquid nitrogen using a chilled mortar and pestle and then homogenized in 5 mL extraction buffer containing of 50 mM phosphate buffer (pH 7.8), 0.1 mM ethylenediaminetetraacetic acid (EDTA), 0.3% (v/v) Triton X-100, and 4% gpolyvinylpyrrolidone (PVP). The homogenate was then immediately centrifuged for 20 min at 12,000 rpm. Phosphate buffer was subsequently added to the supernatant, which was used to analyse the activity of antioxidant enzymes. All operations were carried out at 0–4 °C.

Superoxide (SOD) activity was measured according to its ability to inhibit the photochemical reduction of nitro blue tetrazolium (NBT), which was recorded at 560 nm. One unit of SOD was defined as the amount of enzyme that inhibited NBT reduction by 50%^52^.

Catalase (CAT) activity was measured according to its ability to consume the amount of H_2_O_2_^53^. The CAT reaction solution comprised 50 mM phosphate buffer (pH 7.8), 0.1 M H_2_O_2_ and 0.1 mL enzyme extract. Changes in the absorbance of the reaction solution at 240 nm were recorded for 5 min. One unit of CAT activity was defined as the absorbance change of 0.01 units per minute.

Peroxidase (POD) activity was determined using guaiacol as the substrate and assayed according to the method of Zheng et al*.*^54^ with minor modifications. First, 0.2 mL of the enzyme extract, 2 mL of 10 mM Na-phosphate buffer (pH 7.4), 0.1 mL of 1% guaiacol(v/v) , and 0.1 mL 0.3% H_2_O_2_ (v/v) were mixed together, after which the homogenates were centrifuged at 14,000 rpm for 20 min. The change in absorbance was measured at 470 nm for 5 min via a UV–visible spectrophotometer (T6, Persee, Beijing, P. R. China). One unit of POD activity was defined as an absorbance change of 0.01 units per minute.

In total, 0.5 g of seedling tissue was homogenized at 4 °C in 1 mL of extraction buffer [50 mM potassium phosphate buffer (pH 7.0), 1% Triton X-100, and 7 mM 2-mercaptoethanol] with mortar and pestle. The homogenate was then centrifuged at 25,000 rpm for 20 min, after which the supernatant was used as the crude extract for the ascorbate peroxidase (APX) and glutathione reductase (GR) assay^55^.

A 3 mL reaction mixture was composed of 50 mM phosphate buffer (pH 7.0) that consisted of 0.1 mM EDTA, 0.3 mM ascorbic acid (AsA), 0.06 mM H_2_O_2_ and 0.2 mL of enzyme extract. The homogenate was then centrifuged at 10,000 rpm for 20 min .The oxidation of ascorbate was followed by the decrease in the absorbance at 240 nm^55^.

GR activity was assayed by measuring the decrease in absorbance at 340 nm due to the oxidation of reduced nicotinamide adenine dinucleotide phosphate (NADPH)^55^. A 3 mL reaction mixture comprised 50 mM phosphate buffer (pH 8.2) that consisted of 1 mM EDTA, 0.2 mM NADPH, 1 mM oxidized glutathione (GSSG) and 0.2 mL of enzyme extract.AsA content was determined according to the method of Li et al*.*^56^ with minor modifications. Standards for AsA were prepared in the range of 0 ~ 60 μg in 1 mL of 5% (w/v) TCA, 1 mL alcohol, 0.5 mL 0.4% H_3_PO_4_-alcohol, 1 mL of 0.5% bathophenanthroline (BP)-alcohol and 0.5 mL of 0.03% FeCl_3_-alcohol at 30 °C for 60 min and measured at 534 nm. One gram of leaf tissue was ground in a chilled mortar and pestle in 5 mL of 5% (w/v) TCA. The homogenates were centrifuged at 4000 rpm for 10 min at 4 °C. The supernatant (1 mL) and 1 mL of 5% (w/v) TCA were mixed together according to the method of generating a standard curve. For each sample the AsA content was estimated as described above. To determine the reduced glutathione (GSH) content, 0.1 g frozen leaf tissue was ground in a chilled mortar and pestle with 3 mL of 5% (v/v) TCA. The homogenates were subsequently centrifuged at 2500 rpm for 15 min at 4 °C. The supernatants were then analyzed for their content of GSH^57^. Changes in absorbance of the reaction mixture were measured at 420 nm.

### Measurement of Si content

Si content of maize seedlings was determined colorimetrically by the molybdate blue method and the absorbance was observed at 630 nm. Different amounts of silicic acid were included as standards for determining Si content of plant samples^25^.

### Scanning electron microscopy-based measurements of the stomata

The leaf samples were cut into pieces of approximately 2 × 5 mm in size and fixed for more than 1.5 h with 2.5% glutaraldehyde in 0.1 M phosphate-buffered saline (PBS; pH 6.8) solution. The fixed samples were washed three times with the same solution for 10 min each. Afterward, the samples were dehydrated one time in a graded ethanol series (50%, 70%, and 90%) and two times in absolute ethanol for 10 min. The samples were displaced miscible liquids (comprising a 1:1 mixture of tertiary butanol and absolute ethyl alcohol) and absolute ethyl tertiary butanol for 15 min. The samples were maintained at − 20 °C for 30 min and dried in a freeze-drier (ES-2030, Hitachi, Tokyo, Japan) for 4 h. The observed samples faced up and were adhered to the scanning electron microscope sample table with conductive tape. The sample surfaces were coated with 100–150 Å metal films by ion sputter (E-1010, Hitachi, Tokyo, Japan). The sections were mounted on the sample table, observed with a scanning electron microscope and imaged(S-3400N, Hitachi, Tokyo, Japan).

**Figure 7:**
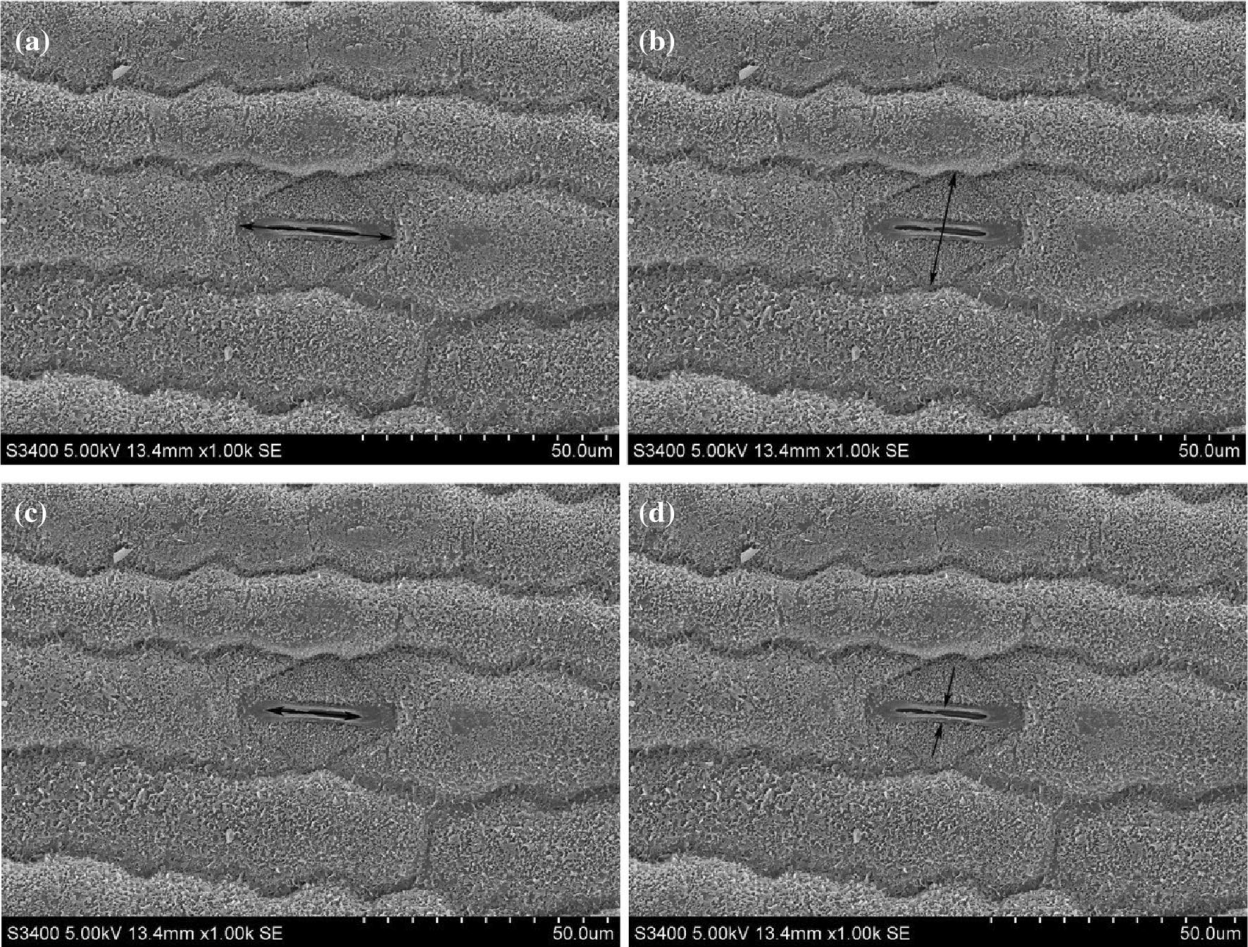
Measurement of stomatal length (**a**), width (**b**), aperture length (**c**) and width (**d**).

The size and aperture of stomata (n = 20) in the center of the lower epidermis of the second fully expanded leaf from the top of four plants in each treatment were observed and imaged via a scanning electron microscopy at 2000 × magnification (Fig. 7).

### Statistical analysis

The data were recorded and processed using Excel 2010 (Microsoft Inc., WA, Redmond, USA) and SPSS 19.0 software (SPSS Inc., Chicago, IL, USA). The variance and significance of the data were analysed. To detect significance differences between the means, Duncan's multiple range test was performed at a significance level of P < 0.05^58^.

## Data Availability

The datasets generated during and/or analyzed during the current study are available from the corresponding author on reasonable request.
